# Cortical thickness abnormalities in long-term remitted Cushing’s disease

**DOI:** 10.1038/s41398-020-00980-6

**Published:** 2020-08-21

**Authors:** S. E. E. C. Bauduin, Z. van der Pal, A. M. Pereira, O. C. Meijer, E. J. Giltay, N. J. A. van der Wee, S. J. A. van der Werff

**Affiliations:** 1 Department of Psychiatry, Leiden University Medical Center (LUMC), Leiden, The Netherlands; 2 Leiden Institute for Brain and Cognition, Leiden, The Netherlands; 3 Department of Endocrinology and Metabolic Diseases and Center for Endocrine Tumors, Leiden University Medical Center, Leiden, The Netherlands

**Keywords:** Neuroscience, Diseases

## Abstract

Long-term remitted Cushing’s disease (LTRCD) patients commonly continue to present persistent psychological and cognitive deficits, and alterations in brain function and structure. Although previous studies have conducted gray matter volume analyses, assessing cortical thickness and surface area of LTRCD patients may offer further insight into the neuroanatomical substrates of Cushing’s disease. Structural 3T magnetic resonance images were obtained from 25 LTRCD patients, and 25 age-, gender-, and education-matched healthy controls (HCs). T1-weighted images were segmented using FreeSurfer software to extract mean cortical thickness and surface area values of 68 cortical gray matter regions and two whole hemispheres. Paired sample *t* tests explored differences between the anterior cingulate cortex (ACC; region of interest), and the whole brain. Validated scales assessed psychiatric symptomatology, self-reported cognitive functioning, and disease severity. After correction for multiple comparisons, ROI analyses indicated that LTRCD-patients showed reduced cortical thickness of the left caudal ACC and the right rostral ACC compared to HCs. Whole-brain analyses indicated thinner cortices of the left caudal ACC, left cuneus, left posterior cingulate cortex, right rostral ACC, and bilateral precuneus compared to HCs. No cortical surface area differences were identified. Cortical thickness of the left caudal ACC and left cuneus were inversely associated with anxiety symptoms, depressive symptoms, and disease duration, although certain associations did not persist after correction for multiple testing. In six of 68 regions examined, LTRCD patients had reduced cortical thickness in comparison to HCs. Cortical thickness of the left caudal ACC was inversely associated with disease duration. This suggests that prolonged and excessive exposure to glucocorticoids may be related to cortical thinning of brain structures involved in emotional and cognitive processing.

## Introduction

Cushing’s disease (CD), a rare endocrine disorder that is caused by an adrenocorticotropic hormone (ACTH) producing pituitary adenoma, is the most common etiology of endogenous Cushing’s syndrome (CS^1^). CS is characterized by chronic exposure to glucocorticoid (GC) excess, with the most common cause being exogenous CS as a consequence of pharmacological GC treatment^1^. Hypercortisolism has been associated with severe physical, psychological, and cognitive impairments, resulting in a substantial deterioration in quality of life. Physical symptoms of CD include abdominal weight gain and abnormal fat distribution, acne, thin skin sensitive to bruising, osteoporosis, hirsutism, muscle weakness, delayed wound healing, and gonadal dysfunction^2^. Stress-related disorders such as mania, anxiety, and depression commonly present alongside CD, as does suicidality^3–5^. Cognitive deficits that are commonly experienced by CD patients include deficits in reasoning, verbal learning, language performance, difficulty in concentrating, visual and spatial information processing, and memory impairments^6–9^. These symptoms suggest that prolonged exposure to an excess of cortisol has a detrimental effect on the central nervous system.

Preclinical studies have found that chronically increased GC exposure can cause psychiatric symptomology (e.g., an anxiodepressive-like phenotype in animals^10,11^), which has been linked to structural and functional changes of certain limbic structures, such as the hippocampus and the anterior cingulate cortex (ACC^12^). In line with these preclinical study findings, long-term exposure to high levels of cortisol in (remitted) CD patients has been associated with functional and structural alterations in similar limbic areas^13^. Considering the severe cortisol dysregulation in CD, changes in cortical thickness and surface area are also expected to be observed in regions affected by CD even after biochemical curation, such as the ACC. However, such analyses are lacking to date.

Pituitary corticotroph adenomas are usually detected as microadenomas (<10 mm), because hyperactivity of the hypothalamic–pituitary–adrenal (HPA-) axis leads to the rapid manifestation of the clinical symptoms of CD^1^. HPA-axis activity is regulated by limbic structures, such as the amygdala, the hippocampus, and the ACC^14^. Under the influence of the circadian rhythm and exposure to stressors, hypothalamic corticotropin-releasing hormone (CRH) secretion stimulates pituitary ACTH secretion. This, in turn, stimulates GC production by the adrenal glands. In healthy individuals, circulating cortisol inhibits CRH and ACTH secretion through a negative feedback loop, however in patients with CD this physiological control mechanism is impaired due to the autonomous secretion of ACTH by the pituitary adenoma, resulting in unabridged hypercortisolism^15^. First-line treatment for patients with CD is transsphenoidal pituitary surgery^16^. Second-line treatment may include additional neurosurgical intervention, medical therapy, radiation therapy, or bilateral adrenalectomy. These second-line treatments often result in deficiencies in pituitary hormone production (i.e., hypopituitarism), the need for chronic replacement therapy, and adverse long-term prognoses^17^. However, usually cortisol levels normalize after removal of the adenoma, paired with concomitant somatic, cognitive, and emotional symptom reduction^18–22^. Nevertheless, a higher prevalence of psychiatric symptomatology often remains in long-term remitted CD (LTRCD)-patients in comparison to healthy controls (HCs^18,22–24^). A plausible explanation for these persistent symptoms remains unknown as of yet.

Previous studies have reported a reduction in cortical thickness in patients with stress-related disorders such as generalized and social anxiety disorder^25,26^ bipolar disorder^27,28^, and major depressive disorder^29,30^. Two earlier studies investigated cortical thickness in CS patients and HCs: the first found no differences in cortical thickness^31^ and the second reported increased cortical thickness in the lateral orbitofrontal and superior frontal cortex in children with CS in compared to HCs, however this study did not adjust for multiple comparisons^32^.

Reductions in gray matter volume of the cingulate, frontal and orbitofrontal cortices, hippocampus, amygdala, inferior temporal gyrus, and striatum have been reported for stress-related disorders such as depression, anxiety and obsessive–compulsive disorder, which are also associated with cortisol dysregulation, albeit on a much smaller scale than in CD^33–37^. In CS patients, earlier studies have found loss of brain volume (for example, in the hippocampus, bicaudate, and third ventricle), which were partially reversible upon biochemical remission^21,38,39^. Analyses conducted in the same cohort of LTRCD patients as the present study have previously revealed reductions in white matter integrity throughout the brain in addition to altered resting-state connectivity between the limbic system and the subgenual ACC in comparison to HCs^24,40^. Furthermore, subcortical gray matter alterations in this patient population were examined using FSL’s integrated registration and segmentation tool (FIRST). There were no differences in gray matter volume or shape for any subcortical regions, however reductions of ACC volumes were found^18^. As FSL’s FIRST uses a similar segmentation approach of the subcortical regions as that of FreeSurfer, subcortical regions were not further examined in this study. The ACC is an area that has been found to remain affected upon curation of CD. Subregions of the ACC are considered critical in cognitive processing of fear and anxiety, cognitive control, emotional functioning, and reward-based decision making; damage to this region may lead to reductions in motivation, spontaneity, and problem-solving capacity, as well as increased apathy and verbalization^18,41–43^. These findings suggest that alterations in structure and connectivity in the brain, and in particular the ACC, may explain part of the cognitive and psychiatric symptoms commonly observed both in active and remitted CD patients.

Two frequently used measures for gray matter analysis are cortical thickness, which is indicative of neuron and glia size, number, and arrangement in specific cortical regions^44–46^ and cortical surface area, which is related to the number of columns in a region of interest^45–47^. Cortical thickness and surface area together constitute gray matter volume, but separately they provide more detailed information on changes in cortical structures. Therefore, cortical thickness and surface area are suggested to be of more etiological relevance than gray matter volume alone^48,49^.

In the present case-control study, our primary objective was to investigate whether LTRCD patients present differences in cortical thickness and surface area in comparison with HCs. We hypothesized that reductions in cortical thickness and changes in surface area of the ACC would be associated with LTRCD. In addition, our secondary objective was to conduct an explorative whole-brain analysis in order to detect possible structural alterations in regions besides the ACC. Moreover, this study aimed to explore associations between structural alterations and measures of psychiatric symptomatology, self-reported cognitive functioning, disease duration, disease severity, and duration of remission. Here, we hypothesized that smaller cortical thickness and surface area would be associated with higher scores on scales assessing psychopathology, lower self-reported cognitive functioning scores, longer disease duration, and/or higher disease severity, and shorter duration of remission.

## Methods

### Subjects

All 49 LTRCD patients (aged 18–60) who were under chronic surveillance at the Leiden University Medical Center (LUMC) were invited either by letter or by telephone to participate in the study. The response rate was 96%. Of these 49 patients, 16 patients (33%) declined to participate with the (f)MRI part of the protocol. Therefore, 31 patients were screened for eligibility. Of these 31 patients, six were excluded due to one of the following exclusion criteria: neurological problems, magnetic resonance imaging (MRI) contraindications, a (history of) drug or alcohol abuse, and/or left handedness. HCs were recruited via advertisements in grocery stores and internet, and were matched pairwise to each patient based on gender, age (between 18 and 60 years), and level of education. A further exclusion criterion regarding the HCs group was a history or presence of a psychiatric disorder. A study aimed at determining sample size estimates for cross-sectional cortical thickness studies using FreeSurfer software (the same processing stream as in the current study), found that a sample of 14 subjects per group are required to detect a thickness difference of 0.6 mm over 95% of the cortical surface using two-sided *t* tests (30 mm FWHM, power = 0.95, *α* = 1.22 × 10^−4 ^^50^). The final sample of the current study consisted of 25 LTRCD patients and 25 matched HCs.

The diagnosis of active CD was confirmed using international guidelines and multiple positive test outcomes, such as increased urinary cortisol excretion rates, decreased overnight suppression by dexamethasone (1 mg), and increased midnight salivary cortisol values. The detailed criteria have previously been published elsewhere^22^. All patients underwent transsphenoidal surgery, after which biochemical cure was confirmed using multiple test outcomes, such as normal overnight suppression of plasma cortisol levels (<50 nmol/l) by dexamethasone (1 mg), normal 24 h urinary cortisol excretion rates (<220 nmol/24 h) and normal cortisol response to CRH stimulation test or insulin tolerance test (>500 nmol/l), indicating hydrocortisone independency. Patients with remaining GC dependency (*n* = 13; 52%), received hydrocortisone replacement (on average 20 mg/day, divided over three doses), and were evaluated twice yearly. Persistent biochemical cure of CD was documented as normal levels for abovementioned diagnostic tests before participation in the study. Duration of disease was defined as the moment earliest somatic signs were presented in the patient’s history and duration of remission was calculated from the date of curative transsphenoidal surgery, or in case of persistent disease, from the date of normalization of biochemical tests after postoperative radiotherapy (mean: 11.2, SD: 8.2, range 0.8–29.3 years after biochemical remission). Further detailed information on patient inclusion and characteristics have previously been published^18^. Patient characteristics and demographics are reported in Table 1, which are identical to the previously published data by Andela et al.^18^. All participants provided written informed consent, and patient and treatment characteristics were obtained from patient records. The study protocol was approved by the medical ethics committee of the LUMC, and is in accordance with the principles of the declaration of Helsinki.

**Table 1:** Demographics and psychometric data of LTRCD patients and matched healthy controls. Data are presented as mean ± standard deviation or number (%), with a significance level set at *P* < 0.05.

	CD patients (*n* = 25) Mean ± SD	Matched controls (*n* = 25) Mean ± SD	*P* value
Gender (female)	21 (84%)	21 (84%)	1.000^a^
Age (years)	45 ± 8	47 ± 7	0.471^b^
Education			0.946^a^
Low	6 (24%)	6 (24%)	
Medium	12 (48%)	11 (44%)	
High	7 (28%)	8 (32%)	
Intracranial volume	1.45 × 10^6^ ± 0.163 × 10^6^	1.48 × 10^6^ ± 0.145 × 10^6^	0.716^b^
MADRS	6.3 ± 5.5	1.4 ± 1.8	<0.0001^c^
Inventory of depressive symptomatology	46.8 ± 13.0	36.3 ± 5.8	0.005^c^
Beck anxiety inventory	28.4 ± 5.7	24.0 ± 3.1	0.003^c^
Fear questionnaire	24.5 ± 17.4	14.2 ± 10.0	0.051^b^
Agoraphobia subscale	6.1 ± 7.9	3.4 ± 4.7	0.477^c^
Blood injury phobia subscale	6.2 ± 8.3	3.2 ± 4.1	0.118^c^
Social phobia subscale	12.2 ± 8.0	7.6 ± 4.9	0.034^b^
Irritability scale	12.1 ± 8.7	8.0 ± 6.1	0.066^c^
Total score > 14	9 (36%)	6 (24%)	
Apathy scale	13.6 ± 6.6	7.8 ± 3.8	0.002^c^
Total score > 14	11 (44%)	2 (8%)	
Cognitive failures questionnaire	38.0 ± 16.5	27.6 ± 9.7	0.023^b^
Disease duration (years)	7.9 ± 7.9		
Duration of remission (years)	11.2 ± 8.2		
Cushing’s syndrome severity index			
Active phase (total)	8.1 ± 2.0		
Remission phase (total)	2.5 ± 1.5		

*MADRS* Montgomery–Åsberg Depression Rating Scale.

^a^*P* values were tested with *X*^2^ test.

^b^*P* values were tested with independent samples *t* test.

^c^*P* values tested with Mann–Whitney *U* test.

### Psychopathological and clinical severity assessments

Psychopathology and self-reported cognitive functioning were assessed using various scales, for which higher sum scores indicate greater symptom severity. The Montgomery–Åsberg Depression Rating Scale (MADRS^51^), and the Inventory of Depression Symptomatology (IDS^52^) were used to assess the severity of depressive symptoms. The MADRS was assessed by the interviewer, whereas all other scales used were self-report. Anxiety was evaluated using the Beck Anxiety Inventory (BAI^53^), and phobic anxiety was measured using the total scores, as well as the agoraphobia, blood injury phobia, and social phobia subscales, of the Fear Questionnaire (FQ^54^). The Irritability Scale and the Apathy Scale were used to assess the severity of irritability and apathy, respectively^55,56^. For both questionnaires, participants were considered to be irritable or apathetic if they present a total score of 14 points or more. Failures in motor function, perception and memory were assessed using the self-report Cognitive Failure Questionnaire (CFQ^57^).

Symptom severity during active and remitted disease state were estimated using the Cushing’s Syndrome Severity Index (CSI^58^). The CSI score during active disease was estimated retrospectively, whereas the score during remission was based on the last annual evaluation. In the analyses, the total CSI score was used for both active and remitted disease state. Scores on this index can range between 0 and 16, with a higher total score indicating greater symptom severity. The information required to score the CSI was obtained from the patient’s clinical history and medical records. The index was scored by two independent raters that reached consensus in case of discrepancy.

### MRI data acquisition

Structural magnetic resonance images were acquired using a Philips 3T system (Philips Healthcare, Best, The Netherlands; software version 3.2.1) at the LUMC. A SENSE-32 channel headcoil was used for transmission and reception of radio frequencies. A sagittal 3D gradient-echo T1-weighted sequence (echo time = 4.6 ms, repetition time = 9.8 ms, 140 slices, scan duration 4:56 min, matrix size = 256 × 256, voxel size = 1.17 × 1.17 × 1.2 mm) was used to acquire anatomical images, which were examined by a neuroradiologist who was blinded for patient details. No macroscopic abnormalities were detected other than age-related white matter intensities and effects of post-transsphenoidal surgery.

### Statistical analyses

Parcellation of 68 (34 left and 34 right) cortical gray matter regions as well as extraction of two whole-hemisphere measures were performed using FreeSurfer (version 5.3.0). A visual quality check and statistical outlier assessment of the 68 regions and two whole-hemisphere measures were performed by two independent individuals according to the ENIGMA imaging protocols (http://enigma.ini.usc.edu/protocols/imaging-protocols/).

All statistical evaluations were performed using IBM SPSS Statistics for Windows version 24 (IBM Corp. Armonk, N.Y., USA) and figures were created using the r package “ggpmisc” as an extension to the package “ggplot2”. We examined differences in cortical thickness and surface area for predetermined regions of interest (ROI): the rostral ACC and the caudal ACC. Moreover, a whole-brain analysis was performed to detect possible unpredicted differences in cortical thickness and/or surface area between LTRCD patients and HCs. Linear regression was performed with intracranial volume (ICV) as an independent variable and the unstandardized residuals were saved. Restructuring of the dataset to a wide format allowed for calculation of the difference between the residuals of patients and controls, (i.e., delta residuals), per region. The assumption of normal distribution of the delta residuals was tested using the Kolmogorov–Smirnov test, boxplots, histograms and normal and detrended normal Q–Q plots. Next, pairwise group-level comparisons between LTRCD patients and controls were performed using pairwise *t* tests (comparing each patient with its matched control) and Wilcoxon signed rank tests using the residuals of cortical thickness and surface area measures. The reported *p* values for the ROIs and the whole-brain analyses are two tailed. All analyses were corrected for multiple testing using the Benjamini–Hochberg procedure^59^, with the false discovery rate (FDR) set at 5% for 70 measures (68 cortical regions and two whole-hemisphere measures) using Cohen’s *d* as a measure of effect size.

Finally, within the LTRCD-patient group, we investigated whether cortical thickness and surface area measures of regions that showed significant differences in the ROI and whole-brain analyses correlated with measures of psychiatric symptom severity, self-reported cognitive functioning, and clinical severity. The questionnaires used for psychopathological assessment show considerable overlap, therefore correction for multiple testing using the Benjamini–Hochberg method with an FDR set at 5% was considered too stringent. Therefore, we corrected for multiple testing using an FDR set at 20%. We report the uncorrected Pearson’s correlations for normally distributed data, and the Spearman’s rho for data that is not normally distributed.

## Results

### Participant characteristics

As previously reported in Andela et al.^18^ the LTRCD-patient group did not differ from the HC group in age, gender, and education. The groups also did not differ significantly in intracranial volume (ICV). Mean disease duration was 7.9 ± (SD) 7.9 years (range 0.8–29.3), and mean duration of remission was 11.2 ± 8.2 years (range 0.8–37.0). Mean CSI score was 8.1 ± 2.0 in active CD and 2.5 ± 1.5 in LTRCD patients at the time of assessment. Compared with HCs, LTRCD patients had significantly higher scores on the MADRS and IDS (MADRS: *p* < 0.001, IDS: *p* = 0.005), the BAI (*p* = 0.003), the social phobia subscale of the FQ (*p* = 0.034), the AS (*p* = 0.002), and the CFQ (*p* = 0.023), and the total FQ score approached significance (*p* = 0.051). There were no significant differences between groups regarding scores on the IS, FQ agoraphobia, and blood injury phobia subscales (Table 1; all demographic and participant characteristics have been previously been reported in Andela et al.^18^).

### ROI analyses

With regard to the ROI analyses, LTRCD patients showed smaller cortical thickness of the left caudal ACC (*p* = 0.002) and the right rostral ACC (*p* = 0.003) compared with HCs. Cohen’s *d* was 0.68 and 0.65 for the left caudal and right rostral ACC respectively, indicating medium effect sizes (see Table 2 and Appendix I for a visual representation). Closer examination of the findings revealed that patients had 6% smaller left caudal ACC thickness and 5% smaller right rostral ACC thickness. ROI surface area analyses revealed no significant differences between LTRCD patients and HCs (see Table 2 and Appendix I for a visual representation).

**Table 2:** ROI analysis of cortical thickness and surface area measures.

Measure	Region	Mean (S.E.)			Δ (S.E.)	Uncorrected *p* value	Cohen’s *d*
		*N*	Cushing’s disease	Matched controls			
Cortical Thickness (mm)	L caudal ACC	25	2.78 (0.03)	2.95 (0.04)	0.18 (0.05)	0.002*	0.68
	L rostral ACC	22	2.93 (0.04)	2.89 (0.03)	−0.04 (0.04)	0.375	−0.19
	R caudal ACC	25	2.74 (0.05)	2.78 (0.06)	0.04 (0.06)	0.541	0.11
	R rostral ACC	25	2.96 (0.04)	3.11 (0.03)	0.15 (0.05)	0.003*	0.65
Surface area (mm^2^)	L caudal ACC	25	534.8 (26.24)	546.1 (21.12)	11.3 (32.6)	0.610	0.10
	L rostral ACC	22	741.4 (25.78)	803.0 (36.06)	61.6 (42.3)	0.119	0.35
	R caudal ACC	25	665.6 (22.61)	652.1 (23.86)	−13.5 (30.3)	0.729	−0.07
	R rostral ACC	25	589.6 (20.53)	560.6 (25.70)	−28.9 (38.9)	0.467	−0.15

*ACC* anterior cingulate cortex.

*Remains significant after Benjamini–Hochberg correction (FDR = 5%) for four comparisons.

### Whole-brain analyses

**Fig. 1:**
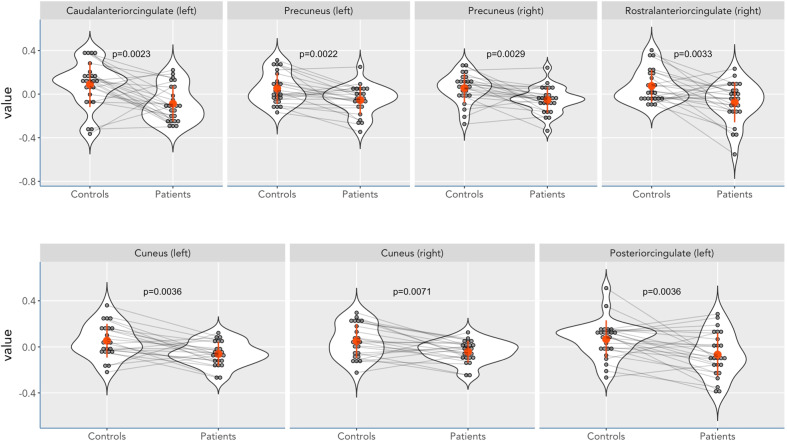
Violin plots of significant cortical thickness differences between LTRCD patients and HCs. Violin plots of cortical thickness measures representing areas that remained significant after Benjamini–Hochberg correction (FDR = 5%) with the exception of the right cuneus.

In comparison to the HC group, LTRCD patients presented smaller cortical thickness of the left caudal ACC (*p* = 0.002), left cuneus (*p* = 0.004), left posterior cingulate cortex (*p* = 0.004), left superior frontal cortex (*p* = 0.041), left supramarginal cortex (*p* = 0.044), right cuneus (*p* = 0.007), right pars opercularis (*p* = 0.037), right rostral ACC (*p* = 0.003), and bilateral precuneus (left: *p* = 0.002, right: *p* = 0.003). However, after correction for multiple testing using the Benjamini–Hochberg method (FDR = 5%) for 70 comparisons, only the differences in the left caudal ACC, left cuneus (Cohen’s *d* = 0.68), left posterior cingulate cortex (Cohen’s *d* = 0.68), right rostral ACC, left precuneus (Cohen’s *d* = 0.70), and right precuneus (Cohen’s *d* = 0.66), remained significant (Table 3 and Fig. 1; see Appendix II for a complete overview). Closer examination of the data revealed that patients had 6% smaller thickness of the left cuneus, 5% smaller thickness of the left posterior cingulate cortex, and 4% smaller thickness of the bilateral precuneus.

**Table 3:** Whole-brain analysis of cortical thickness measures.

Region	Mean (S.E.)			Δ (S.E.)	Uncorrected *p* value	Cohen’s *d*
	*N*	Cushing’s disease	Matched controls			
L caudal ACC	25	2.78 (0.03)	2.95 (0.04)	0.18 (0.05)	0.002*	0.68
L precuneus	24	2.34 (0.03)	2.45 (0.03)	0.10 (0.03)	0.002*	0.70
R precuneus	25	2.35 (0.02)	2.45 (0.03)	0.11 (0.03)	0.003*	0.66
R rostral ACC	25	2.96 (0.04)	3.11 (0.03)	0.15 (0.05)	0.003*	0.65
L cuneus	23	1.71 (0.02)	1.82 (0.03)	0.11 (0.04)	0.004*	0.68
R cuneus	23	1.76 (0.02)	1.85 (0.03)	0.09 (0.03)	0.007	0.65
L posterior cingulate	25	2.46 (0.04)	2.60 (0.03)	0.13 (0.04)	0.004*	0.68

*ACC* anterior cingulate cortex.

*Remains significant after Benjamini–Hochberg correction (FDR = 5%) for 70 comparisons.

Whole-brain analyses of surface area measures revealed greater surface area of the right banks of the superior temporal sulcus (*p* = 0.011). However, this difference did not remain significant after correction for multiple testing.

### Correlation analyses

**Fig. 2:**
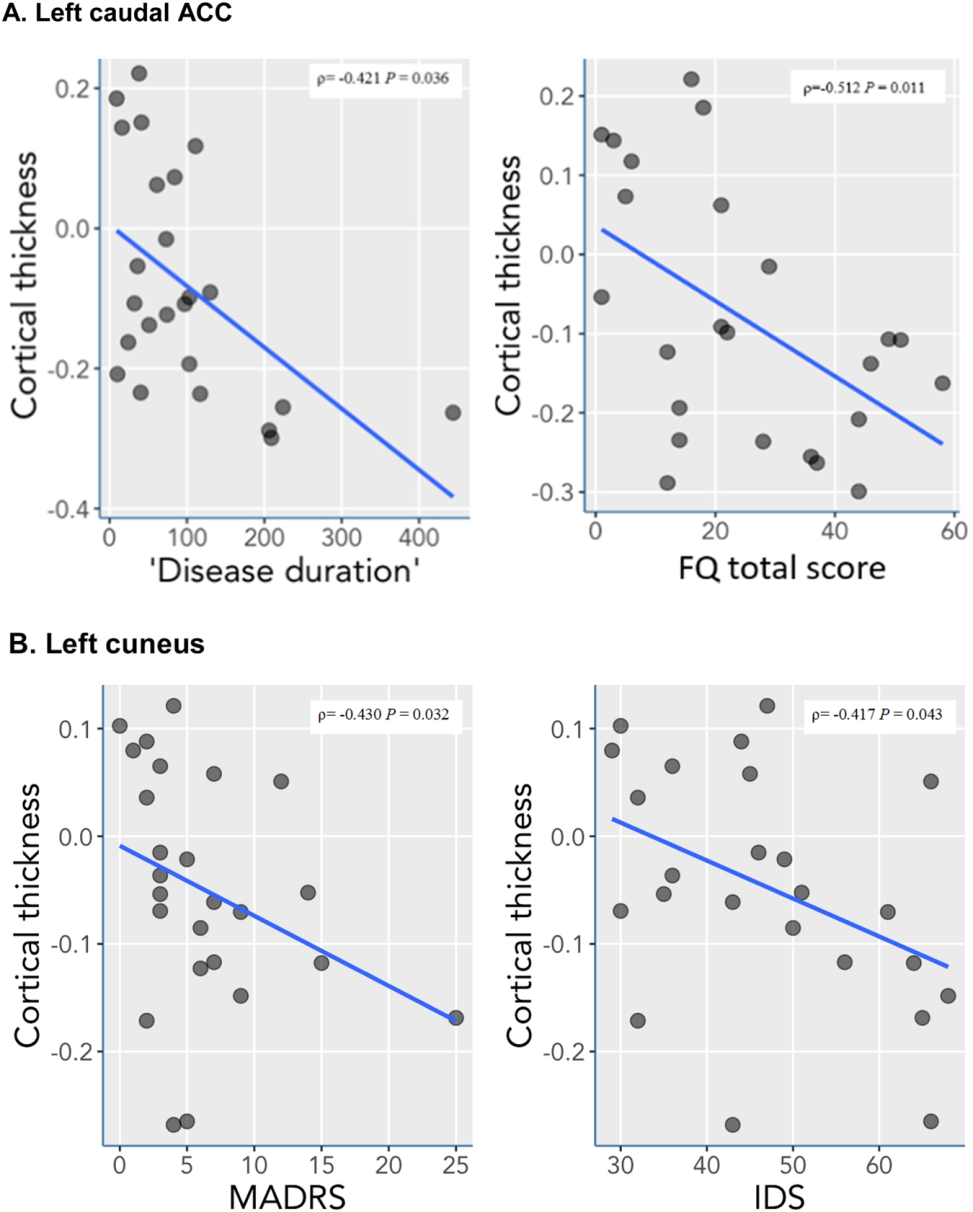
Significant correlations between cortical thickness measures and psychometric data in LTRCD patients. **a**, **b** Significant correlations after Benjamini–Hochberg correction (FDR = 20%) for 11 comparisons between cortical thickness of the left caudal ACC and disease duration in months and total FQ scores (**a**), and significant correlations prior to adjusting for multiple comparisons between cortical thickness of the left cuneus and the MADRS and IDS (**b**).

Correlation analyses were run for brain regions that were found to be significantly different in LTRCD patients in comparison to HCs with the psychopathology and clinical severity assessments. Within the LTRCD group, cortical thickness measures of the left caudal ACC were significantly negatively associated with disease duration (*r* = −0.421, *p* = 0.036). Considering that the total FQ score between the groups approached significance (*p* = 0.051) and the mean total FQ score between the groups differed more than 10 points, indicating a clinically relevant difference, associations between the FQ psychopathology (sub)scales and these brain regions were further investigated. Pertaining to this, cortical thickness measures of the left caudal ACC and the total FQ score were found to be negatively associated (*r* = −0.512, *p* = 0.011; see Fig. 2a and Appendix III for further details). Cortical thickness of the left cuneus was significantly negatively associated with scores on the MADRS (*r* = −0.430, *p* = 0.032) and the IDS (*r* = −0.417, *p* = 0.043; see Fig. 2b and Appendix IV). After correcting for multiple comparisons using the Benjamini–Hochberg method (FDR = 20%) for 11 tests, associations between the left caudal ACC with disease duration and the total FQ score remained statistically significant. No other significant associations between cortical thickness measures and scores on psychopathology scales, measures of disease duration, duration of remission, and clinical disease severity were found (Appendices V–VIII).

## Discussion

This study aimed to investigate whether LTRCD patients and matched HCs differ in cortical thickness and/or surface area. We found smaller cortical thickness in several key regions for emotional and cognitive processing: the left caudal and right rostral ACC, the left cuneus, left posterior cingulate cortex, and bilateral precuneus in LTRCD patients compared with controls, while no significant differences in surface area between the groups were observed. Furthermore, correlation analyses within the patient group indicated that cortical thickness of the left caudal ACC was inversely associated with disease duration and total FQ score, although group differences on the total FQ score did not fully meet the significance threshold. Prior to adjusting for multiple comparisons, cortical thickness of the left cuneus was inversely associated with sum scores on the MADRS and IDS.

Our hypothesis that LTRCD patients present more thinning of the ACC in comparison with HCs was confirmed. These findings are not in line with two previous studies investigating cortical thickness in CS patients. One study did not identify any differences in cortical thickness^31^, and the second study identified increased cortical thickness in the lateral orbitofrontal- and superior frontal cortex in children with CS in compared to HCs but did not adjust for multiple comparisons^32^. As the differences found in these areas appear to be marginal, it is unlikely that these effects would have persisted after correction. A possible explanation why these earlier studies did not find results in line with ours could be due to the differences between the CS patient populations included in the studies (i.e., our study consisted solely of patients with remitted CD and the other studies also included patients with other causes of CS), or perhaps due to differences in FreeSurfer versions (e.g., the current study used a newer version than that denoted in the Crespo et al.^31^ paper (v5.3.0 versus v4.3.1). Unfortunately, the Tirosh et al.^32^ paper did not indicate the version of FreeSurfer they used. Using the more recent releases has been posited to provide a more accurate segmentation, although differences may also be caused due to different software builds^60^. Our findings are in accordance with previous findings of Andela et al.^13^ who in the same cohort of LTRCD patients and matched HCs observed reductions in gray matter volume of parts of the bilateral ACC in LTRCD patients.

Furthermore, our results are also in line with findings from an earlier animal study, where reductions in ACC volumes were observed in rats exposed to a GC excess^12^. Limbic structures such as the ACC, hippocampus, and amygdala critically control the activity of the HPA axis^14^. These regions express high levels of glucocorticoid receptors (GR) and mineralocorticoid receptors (MR), making them vulnerable to GC excess as seen in stress-related disorders and more severely in CD. Interestingly, GR and MR are prevalent throughout the brain and not solely in regions affected in LTRCD. The enzyme 11β-HSD2 protects MR and GR from GC excess by converting cortisol into the inactive metabolite cortisone. However, 11β-HSD2 is not expressed in limbic structures such as the ACC, allowing for MR and GR activation in this region^61,62^. It is also a possibility that structural changes are mediated by transsynaptic mechanisms.

Volume changes may reflect changes in any population of neuronal or nonneuronal glia cells in the affected areas, all of which likely are GC sensitive to some extent. Previous studies have repeatedly shown that dendrites, spines, and expression of synaptic molecules are affected by chronic stress^63–68^. A significant loss of synapses on pyramidal cells of hippocampal region CA3, as well as morphological changes in afferent mossy fibers terminating on these neurons, have been observed in animals exposed to GC excess. Moreover, remodeling of pyramidal cells in the prefrontal cortex was observed as a result of exposure to stress^69^. Such processes may cause damage to white matter tracts, which could explain previous findings of reduced white matter integrity and altered resting-state connectivity in LTRCD patients^40^. Persistent hypercortisolism may ultimately lead to loss of neurons^38^. This has been related to increased synaptic glutamate accumulation, leading to increased stimulation of N-methyl-D-aspartate receptors, and elevated postsynaptic intracellular Ca^2+^ levels^61,70,71^. This increases the susceptibility of postsynaptic neurons to injury and cell death, which may be an underlying cause of smaller cortical thickness as observed in the present study. It has been proposed that loss of brain volume induced by chronic hypercortisolism is likely caused by a combination of the factors described above^61,70^.

Apart from the thinning of the left caudal ACC and right rostral ACC, whole-brain analyses also revealed smaller cortical thickness of the bilateral precuneus, left cuneus, and left posterior cingulate cortex in LTRCD patients. The precuneus plays a critical role in behavioral inhibition, which is implicated in cognitive and emotional functioning^72,73^. Moreover, the precuneus is involved in integration of visual and spatial information with the memory domain^74^. These are functions in which patients with LTRCD often experience persistent deficits^6–8,22^. The cuneus plays a critical role in basic visual processing, in which impairments are commonly experienced by LTRCD patients^6^. The cuneus has also been positively associated with inhibitory control in bipolar patients”^75^. The posterior cingulate cortex is a central node within the default mode network of the brain, and together with the precuneus, has been implicated as a neural substrate for human awareness. Moreover, it has also been posed to have a prominent role in pain, episodic memory retrieval^76^, and working memory performance^77^. In partial concurrence with these findings, an earlier study investigating episodic and working memory in female patients with long-term remitted CS found decreased functional brain response during episodic and working memory tests^78^. Furthermore, the precuneus, cuneus, posterior cingulate cortex, and ACC are located next to one another and show strong reciprocal connectivity, and are involved in the large-scale default mode network^40,74,79^. Given the observed cortical abnormalities of these regions in the present study, these findings may support the hypothesis that structural changes occur through transsynaptic mechanisms.

In contrast to our hypothesis, no differences were observed in cortical surface area between LTRCD patients and HCs. Several previous studies examining cortical thickness and surface area in adults with generalized- and social anxiety disorder and MDD have presented similar findings, namely reductions in cortical thickness of certain brain areas, but with no differences in cortical surface area^25,26,29^. The discrepancies between our findings in cortical thickness and surface area suggest that there are distinct (genetic and biological) pathways that affect these measures. Consistent findings in previous studies indicate that cortical thickness and surface area are genetically independent, with the result that both measures are driven by different cellular mechanisms and have different developmental trajectories^80–83^. Our discrepant findings suggest that alterations in gray matter volume that we previously observed may be explained by changes in cortical thickness alone, without changes in surface area. The null findings in our surface area analyses suggest that cortical thickness may have more etiological value than surface area. However, previous studies have shown that surface area has more influence on gray matter volume than cortical thickness^49,83,84^, although this may differ for patients with hypercortisolism and may thus be condition and context dependent.

Correlation analyses revealed multiple significant negative associations between psychopathology measures and cortical thickness of the left cuneus and the left caudal ACC prior to adjusting for multiple comparisons. The psychopathology measures assessed depressive and anxiety symptoms, which are commonly observed psychopathologies in both patients with active CD and with LTRCD. Prior to adjusting for multiple comparisons, the left cuneus was found to be associated with MADRS and IDS scores. A previous study conducted within the same participant cohort as the present study compared LTRCD patients with HCs in terms of presence and severity of psychopathology and cognitive failure. They reported significantly higher levels of depressive symptoms, anxiety, social phobia, apathy, and cognitive failure in LTRCD patients^18^. These findings also support the hypothesis that depressive symptoms and anxiety in LTRCD patients are associated with structural brain changes^22^. Furthermore, a significant negative correlation between left caudal ACC thickness and disease duration was found, offering further support that prolonged exposure to excessive amounts of cortisol may lead to more severe effects on cortical brain structures. Also, significant negative associations were found between the left caudal ACC and the total FQ score. Interestingly, an earlier study found thinning of the cingulate cortex in spider-phobic patients^42^, indicating that the thinning of this brain region may be related to phobias in general. However, as the present study is cross-sectional, no causal conclusions can be drawn, and thus the possibility that structural alterations were already present before onset of CD should be considered. Nevertheless, further research into these relationships may create possibilities for developing specialized therapies for specific patient groups.

The present study provides a valuable contribution to the existing literature by demonstrating that smaller cortical thickness is at least partially responsible for smaller gray matter volumes of the ACC in patients with LTRCD. A considerable strength of our study was the matching of participants for age, gender and education, allowing for paired analysis without correction for these factors. A second strength is the homogeneity of the patient cohort in terms of treatment. It is, however, important to realize that considerable heterogeneity concerning disease duration and duration of remission still exists, which may reduce the power of the study. Nonetheless, the study had sufficient power to detect a number of structural differences even after Benjamini–Hochberg correction. This exemplifies that transient excessive exposure of cortisol excess can result in long-lasting, and possibly irreversible effects on the human brain.

A limitation of this study were the instruments used for the psychopathological assessment, in particular the CSI and CFQ. Although both have been validated repeatedly, the CSI score during active disease was estimated retrospectively and the CFQ cannot replace an elaborate neuropsychological assessment. This may have resulted in a less accurate estimation of disease severity and cognitive functioning. Next, the program FreeSurfer has difficulty with the parcellation of regions with natural anatomical variation such as the cingulate cortex, especially in the presence of a paracingulate sulcus. This affects the surrounding regions and makes accurate estimation of cortical thickness and surface area measures more challenging, although brain segmentations were quality checked by means of visual inspection by two independent raters, and discrepancies between raters were reassessed by the two parties. Finally, despite the reliable quantification of factors influencing gray matter volume provided by cortical thickness and surface area, these measures do not elucidate the physiological processes involved in volumetric changes. An 1H-MRS study conducted in remitted CS patients revealed alterations in hippocampal Glx (Glutamate + Glutamine), NAA (N-Acetyl-aspartate), and total NAA (N-Acetyl-Aspartate + N-Acetyl-aspartyl-Glutamate^85^). Disrupted Glx balance may result in neuronal damage^86^, while reduced NAA levels indicate neuronal dysfunction or loss^87,88^. Interestingly, these metabolite alterations were observed in absence of gray matter volume changes, suggesting that metabolite alterations may precede structural changes. Similarly, functional abnormalities may also be present despite a lack of structural alterations. Therefore, further research examining functional and biochemical changes is required to increase our understanding of the mechanisms underlying CD.

In conclusion, this study demonstrates that patients with LTRCD present cortical thickness rather than surface area abnormalities, building upon previous knowledge, and highlighting certain brain regions that have not been identified as different in the CD patient population to date. Differences were found in key regions for emotional and cognitive processing compared to HCs, with cortical thinning of the left caudal ACC, left cuneus, left posterior cingulate cortex, right rostral ACC, and bilateral precuneus. Moreover, within the LTRCD group, cortical thickness of the left caudal ACC was negatively associated with disease duration and total FQ score. These findings present a possible explanation for volumetric alterations observed in patients with LTRCD and suggest that longer duration of exposure to GC excess has a more severe effect on brain structures and persisting psychiatric symptomatology. It is important to note that a lateralization effect was found in almost all of the significantly different brain structures, suggesting that the left and right areas of certain cortical brain structures differ in their functionality. Disentangling the specific functionality of these brain regions may lead to further valuable insights into the effects of long-term exposure of cortisol on the brain. Future research using longitudinal study designs to examine functional and physiological changes is required to elucidate the pathways leading to persisting structural alterations in the brain of patients with CD, which may aid in improving treatment and prevention strategies for patients with CD as well as for patients with stress-related disorders^77^.

## Supplementary information

Appendices
