# Supplementary material for: Cortical thickness abnormalities in long-term remitted Cushing’s disease
Source: Transl Psychiatry. 2020 Aug 21;10:293. doi: 10.1038/s41398-020-00980-6 (PMC7443132; doi:10.1038/s41398-020-00980-6)
Supplement: Supplementary file 1 — Appendices [file 41398_2020_980_MOESM1_ESM.docx]

**Appendices**

**Appendix I:**


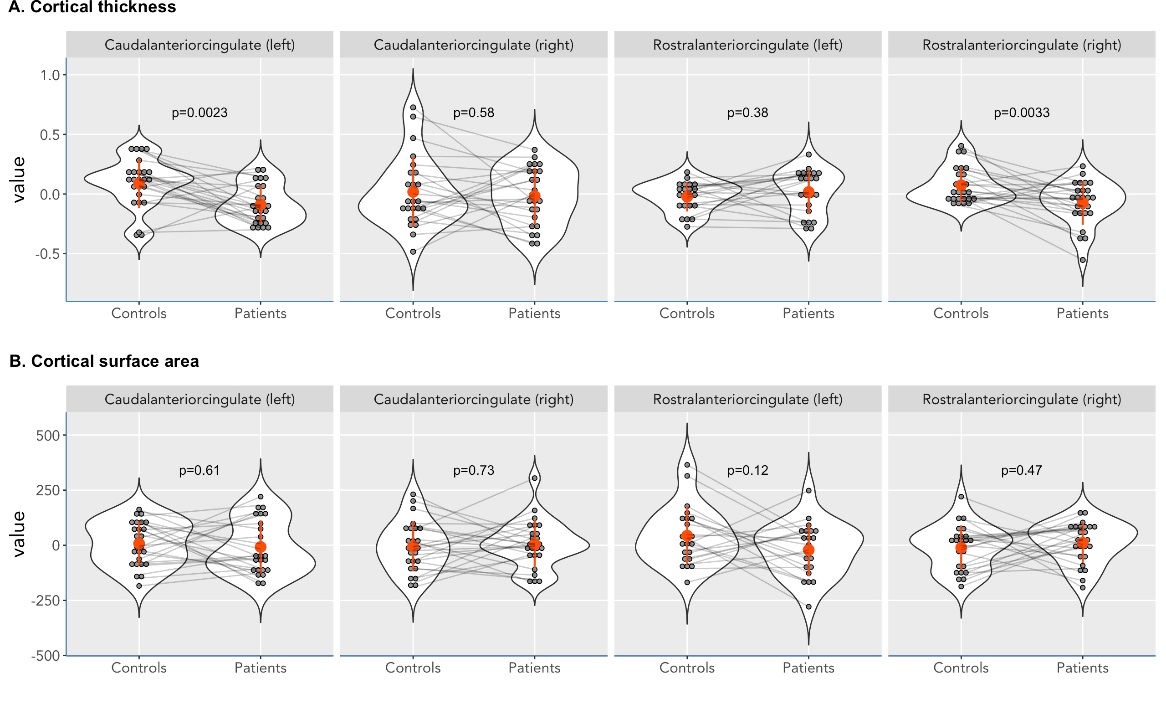


*Figure.* Violin plots of cortical thickness ROIs (A) and surface area ROIs (B)

**Appendix II**: Complete overview of cortical thickness measures for the whole brain.

| **Measure** | **Region** | **Mean (S.E.)** | | | **Δ (mm^2^) (S.E.)** | **Uncorrected p-value** |
| --- | --- | --- | --- | --- | --- | --- |
|  |  | **N** | **Cushing’s Disease** | **Matched controls** |  |  |
| Left | L banks | 16 | 2.49 (0.05) | 2.46 (0.05) | -0.03 (0.08) | 0.680 |
| Cortical | **L caudalanteriorcingulate** | **25** | **2.78 (0.03)** | **2.95 (0.04)** | **0.18 (0.05)** | **0.002** |
| thickness | L caudalmiddlefrontal | 24 | 2.56 (0.03) | 2.63 (0.03) | 0.07 (0.04) | 0.116 |
| (mm^2^) | **L cuneus** | **23** | **1.71 (0.02)** | **1.82 (0.03)** | **0.11 (0.04)** | **0.004** |
|  | L entorhinal | 25 | 3.28 (0.06) | 3.28 (0.06) | 0.01 (0.08) | 0.906 |
|  | L frontalpole | 24 | 2.69 (0.05) | 2.86 (0.06) | 0.16 (0.09) | 0.069 |
|  | L fusiform | 25 | 2.67 (0.03) | 2.64 (0.03) | -0.02 (0.04) | 0.650 |
|  | L inferiorparietal | 25 | 2.45 (0.02) | 2.51 (0.03) | 0.06 (0.03) | 0.079 |
|  | L inferiortemporal | 25 | 2.73 (0.02) | 2.75 (0.02) | 0.02 (0.03) | 0.440 |
|  | L insula | 24 | 2.95 (0.04) | 3.03 (0.03) | 0.09 (0.05) | 0.109 |
|  | L isthmuscingulate | 24 | 2.42 (0.04) | 2.47 (0.03) | 0.04 (0.05) | 0.370 |
|  | L lateraloccipital | 24 | 2.04 (0.02) | 2.09 (0.03) | 0.05 (0.04) | 0.163 |
|  | L lateralorbitofrontal | 25 | 2.70 (0.03) | 2.71 (0.03) | 0.01 (0.04) | 0.726 |
|  | L lingual | 24 | 1.85 (0.02) | 1.90 (0.03) | 0.05 (0.03) | 0.124 |
|  | L medialorbitofrontal | 23 | 2.47 (0.02) | 2.47 (0.03) | 0.004 (0.04) | 0.916 |
|  | L middletemporal | 18 | 2.86 (0.03) | 2.87 (0.04) | 0.008 (0.05) | 0.858 |
|  | L paracentral | 24 | 2.36 (0.05) | 2.44 (0.04) | 0.09 (0.07) | 0.208 |
|  | L parahippocampal | 25 | 2.77 (0.07) | 2.69 (0.04) | -0.08 (0.08) | 0.307 |
|  | L parsopercularis | 25 | 2.59 (0.03) | 2.63 (0.03) | 0.04 (0.04) | 0.382 |
|  | L parsorbitalis | 25 | 2.74 (0.06) | 2.75 (0.04) | 0.01 (0.08) | 0.890 |
|  | L parstriangularis | 24 | 2.44 (0.03) | 2.49 (0.03) | 0.06 (0.04) | 0.117 |
|  | L pericalcarine | 22 | 1.46 (0.02) | 1.49 (0.03) | 0.03 (0.03) | 0.301 |
|  | L postcentral | 24 | 2.03 (0.03) | 2.10 (0.03) | 0.06 (0.05) | 0.183 |
|  | **L posteriorcingulate** | **25** | **2.93 (0.04)** | **2.89 (0.03)** | **0.133 (0.04)** | **0.004** |
|  | L precentral | 23 | 2.51 (0.05) | 2.58 (0.04) | 0.07 (0.06) | 0.225 |
|  | **L precuneus** | **24** | **2.34 (0.03)** | **2.45 (0.03)** | **0.11 (0.03)** | **0.002** |
|  | L rostralanteriorcingulate | 22 | 2.93 (0.04) | 2.89 (0.03) | -0.04 (0.04) | 0.377 |
|  | L rostralmiddlefrontal | 20 | 2.44 (0.02) | 2.44 (0.02) | 0.007 (0.03) | 0.842 |
|  | L superiorfrontal | 24 | 2.76 (0.03) | 2.83 (0.03) | 0.07 (0.33) | 0.041 |
|  | L superiorparietal | 25 | 2.15 (0.03) | 2.21 (0.02) | 0.07 (0.04) | 0.097 |
|  | L superiortemporal | 18 | 2.74 (0.03) | 2.77 (0.03) | 0.04 (0.04) | 0.411 |
|  | L supramarginal | 25 | 2.54 (0.02) | 2.62 (0.03) | 0.07 (0.03) | 0.044 |
|  | L temporalpole | 23 | 3.54 (0.06) | 3.69 (0.06) | 0.14 (0.09) | 0.117 |
|  | L transversetemporal | 25 | 2.27 (0.06) | 2.80 (0.04) | 0.09 (0.07) | 0.185 |
| Right | R banks | 24 | 2.61 (0.04) | 2.57 (0.04) | -0.04 (0.06) | 0.482 |
| Cortical | R caudalanteriorcingulate | 25 | 2.74 (0.05) | 2.78 (0.06) | 0.036 (0.06) | 0.583 |
| thickness | R caudalmiddlefrontal | 25 | 2.58 (0.03) | 2.60 (0.03) | 0.023 (0.04) | 0.568 |
| (mm^2^) | **R cuneus** | **23** | **1.76 (0.02)** | **1.85 (0.03)** | **0.093 (0.03)** | **0.007** |
|  | R entorhinal | 25 | 3.53 (0.06) | 3.41(0.07) | -0.115 (0.09) | 0.218 |
|  | R frontalpole | 25 | 2.78 (0.06) | 2.87 (0.05) | 0.083 (0.08) | 0.312 |
|  | R fusiform | 25 | 2.67 (0.03) | 2.67 (0.03) | 0.006 (0.04) | 0.888 |
|  | R inferiorparietal | 23 | 2.49 (0.02) | 2.54 (0.02) | 0.055 (0.03) | 0.094 |
|  | R inferiortemporal | 25 | 2.75 (0.03) | 2.71 (0.02) | -0.036 (0.03) | 0.198 |
|  | R insula | 24 | 3.04 (0.05) | 3.05 (0.03) | 0.011 (0.06) | 0.843 |
|  | R isthmuscingulate | 24 | 2.48 (0.05) | 2.49 (0.04) | 0.014 (0.07) | 0.840 |
|  | R lateraloccipital | 24 | 2.11 (0.02) | 2.17 (0.03) | 0.061 (0.03) | 0.082 |
|  | R lateralorbitofrontal | 25 | 2.67 (0.03) | 2.67 (0.03) | <0.001 (0.04) | 0.999 |
|  | R lingual | 24 | 1.91 (0.02) | 1.94 (0.03) | 0.034 (0.03) | 0.341 |
|  | R medialorbitofrontal | 24 | 2.56 (0.03) | 2.60 (0.03) | 0.045 (0.04) | 0.281 |
|  | R middletemporal | 25 | 2.89 (0.03) | 2.91 (0.03) | 0.026 (0.04) | 0.517 |
|  | R paracentral | 25 | 2.38 (0.05) | 2.44 (0.04) | 0.058 (0.06) | 0.347 |
|  | R parahippocampal | 25 | 2.70 (0.05) | 2.72 (0.04) | 0.018 (0.06) | 0.770 |
|  | R parsorbitalis | 25 | 2.68 (0.04) | 2.70 (0.04) | 0.014 (0.06) | 0.815 |
|  | R parsopercularis | 24 | 2.55 (0.04) | 2.64 (0.03) | 0.089 (0.04) | 0.037 |
|  | R parstriangularis | 25 | 2.51 (0.04) | 2.51 (0.04) | 0.002 (0.05) | 0.967 |
|  | R pericalcarine | 23 | 1.50 (0.02) | 1.53 (0.02) | 0.035 (0.03) | 0.234 |
|  | R postcentral | 22 | 2.05 (0.03) | 2.08 (0.03) | 0.034 (0.04) | 0.386 |
|  | R posteriorcingulate | 23 | 2.56 (0.03) | 2.57 (0.04) | 0.009 (0.04) | 0.844 |
|  | R precentral | 23 | 2.51 (0.04) | 2.54 (0.04) | 0.029 (0.04) | 0.525 |
|  | **R precuneus** | **25** | **2.35 (0.02)** | **2.45 (0.03)** | **0.108 (0.03)** | **0.003** |
|  | **R rostralanteriorcingulate** | **25** | **2.96 (0.04)** | **3.11 (0.03)** | **0.149 (0.05)** | **0.003** |
|  | R rostralmiddlefrontal | 19 | 2.41 (0.02) | 2.45 (0.03) | 0.040 (0.03) | 0.263 |
|  | R superiorfrontal | 25 | 2.78 (0.03) | 2.82 (0.03) | 0.047 (0.03) | 0.186 |
|  | R superiorparietal | 25 | 2.13 (0.03) | 2.19 (0.03) | 0.058 (0.03) | 0.104 |
|  | R superiortemporal | 24 | 2.79 (0.03) | 2.83 (0.03) | 0.045 (0.05) | 0.347 |
|  | R supramarginal | 22 | 2.57 (0.02) | 2.58 (0.03) | 0.007 (0.03) | 0.839 |
|  | R temporalpole | 22 | 3.72 (0.07) | 3.78 (0.05) | 0.056 (0.08) | 0.503 |
|  | R transversetemporal | 25 | 2.33 (0.06) | 2.39 (0.04) | 0.062 (0.08) | 0.422 |

**Appendix III.** Correlations between cortical thickness of the left caudal anterior cingulate cortex and measures of behavioural and clinical severity within the patient group.

| **Behavioral and clinical severity scales** | **Pearson** | |  | | **Spearman’s rho** | |
| --- | --- | --- | --- | --- | --- | --- |
|  | **Correlation coefficient** | **P value** | | **Correlation coefficient** | | **P value** |
| MADRS |  |  | | 0.159 | | 0.449 |
| Inventory of Depressive  Symptomatology | 0.001 | 0.998 | |  | |  |
| Beck Anxiety Inventory | 0.116 | 0.590 | |  | |  |
| Fear Questionnaire | -0.512 | **0.011*** | |  | |  |
| Social phobia  subscale | -0.287 | 0.174 | |  | |  |
| Apathy Scale | 0.050 | 0.817 | |  | |  |
| Cognitive Failures Questionnaire | -0.021 | 0.924 | |  | |  |
| Disease duration |  |  | | -0.421 | | **0.036*** |
| Duration of remission |  |  | | -0.108 | | 0.608 |
| Cushing’s Syndrome  Severity Index |  |  | |  | |  |
| Active phase | 0.008 | 0.971 | |  | |  |
| Remission phase | 0.036 | 0.863 | |  | |  |

MADRS = Montgomery-Åsberg Depression Rating Scale

* = remains significant after Benjamini-Hochberg correction (FDR=20%) for 11 comparisons.

**Appendix IV.** Correlations between cortical thickness of the left cuneus and measures of behavioural and clinical severity within the patient group.

| **Behavioral and clinical severity scales** | **Pearson** | | **Spearman’s rho** | |
| --- | --- | --- | --- | --- |
|  | **Correlation coefficient** | **P value** | **Correlation coefficient** | **P value** |
| MADRS |  |  | -0.430 | **0.032*** |
| Inventory of Depressive  Symptomatology | -0.417 | **0.043*** |  |  |
| Beck Anxiety Inventory | -0.401 | 0.052 |  |  |
| Fear Questionnaire | -0.143 | 0.505 |  |  |
| Social phobia  Subscale | -0.324 | 0.123 |  |  |
| Apathy Scale | -0.153 | 0.477 |  |  |
| Cognitive Failures Questionnaire | -0.262 | 0.215 |  |  |
| Disease duration |  |  | -0.260 | 0.209 |
| Duration of remission |  |  | 0.112 | 0.593 |
| Cushing’s Syndrome  Severity Index |  |  |  |  |
| Active phase | 0.098 | 0.641 |  |  |
| Remission phase | -0.223 | 0.285 |  |  |

MADRS = Montgomery-Åsberg Depression Rating Scale

* = remains significant after Benjamini-Hochberg correction (FDR=20%) for 11 comparisons.

**Appendix V.** Correlations between cortical thickness of the right rostral anterior cingulate cortex and measures of behavioral and clinical severity within the patient group.

| **Behavioral and clinical severity scales** | **Pearson** | | **Spearman’s rho** | |
| --- | --- | --- | --- | --- |
|  | **Correlation coefficient** | **P value** | **Correlation coefficient** | **P value** |
| MADRS |  |  | 0.067 | 0.749 |
| Inventory of Depressive  Symptomatology | -0.040 | 0.852 |  |  |
| Beck Anxiety Inventory | 0.116 | 0.589 |  |  |
| Fear Questionnaire | -0.052 | 0.809 |  |  |
| Social phobia  subscale | 0.081 | 0.708 |  |  |
| Apathy Scale | 0.291 | 0.168 |  |  |
| Cognitive Failures Questionnaire | -0.147 | 0.493 |  |  |
| Disease duration |  |  | -0.173 | 0.407 |
| Duration of remission |  |  | -0.360 | 0.077 |
| Cushing’s Syndrome  Severity Index |  |  |  |  |
| Active phase | -0.059 | 0.781 |  |  |
| Remission phase | -0.313 | 0.128 |  |  |

MADRS = Montgomery-Åsberg Depression Rating Scale

* = remains significant after Benjamini-Hochberg correction (FDR=20%) for 11 comparisons.

**Appendix VI.** Correlations between cortical thickness of the left posterior cingulate cortex and measures of behavioural and clinical severity within the patient group.

| **Behavioral and clinical severity scales** | **Pearson** | | **Spearman’s rho** | |
| --- | --- | --- | --- | --- |
|  | **Correlation coefficient** | **P value** | **Correlation coefficient** | **P value** |
| MADRS |  |  | -0.103 | 0.626 |
| Inventory of Depressive  Symptomatology | -0.231 | 0.277 |  |  |
| Beck Anxiety Inventory | -0.229 | 0.282 |  |  |
| Fear Questionnaire | -0.207 | 0.333 |  |  |
| Social phobia  subscale | -0.233 | 0.273 |  |  |
| Apathy Scale | 0.012 | 0.956 |  |  |
| Cognitive Failures Questionnaire | -0.343 | 0.101 |  |  |
| Disease duration |  |  | -0.121 | 0.565 |
| Duration of remission |  |  | -0.105 | 0.619 |
| Cushing’s Syndrome  Severity Index |  |  |  |  |
| Active phase | 0.007 | 0.975 |  |  |
| Remission phase | -0.239 | 0.249 |  |  |

MADRS = Montgomery-Åsberg Depression Rating Scale

* = remains significant after Benjamini-Hochberg correction (FDR=20%) for 11 comparisons.

**Appendix VII.** Correlations between cortical thickness of the left precuneus and measures of behavioural and clinical severity within the patient group.

| **Behavioral and clinical severity scales** | **Pearson** | | **Spearman’s rho** | |
| --- | --- | --- | --- | --- |
|  | **Correlation coefficient** | **P value** | **Correlation coefficient** | **P value** |
| MADRS |  |  | -0.175 | 0.402 |
| Inventory of Depressive  Symptomatology | -0.193 | 0.367 |  |  |
| Beck Anxiety Inventory | -0.271 | 0.200 |  |  |
| Fear Questionnaire | -0.173 | 0.419 |  |  |
| Social phobia subscale | -0.381 | 0.066 |  |  |
| Apathy Scale | -0.134 | 0.533 |  |  |
| Cognitive Failures Questionnaire | -0.246 | 0.247 |  |  |
| Disease duration |  |  | -0.206 | 0.323 |
| Duration of remission |  |  | -0.026 | 0.901 |
| Cushing’s Syndrome  Severity Index |  |  |  |  |
| Active phase | 0.325 | 0.113 |  |  |
| Remission phase | -0.212 | 0.310 |  |  |

MADRS = Montgomery-Åsberg Depression Rating Scale

* = remains significant after Benjamini-Hochberg correction (FDR=20%) for 11 comparisons.

**Appendix VIII.** Correlations between cortical thickness of the right precuneus and measures of behavioural and clinical severity within the patient group.

| **Behavioral and clinical severity scales** | **Pearson** | | **Spearman’s rho** | |
| --- | --- | --- | --- | --- |
|  | **Correlation coefficient** | **P value** | **Correlation coefficient** | **P value** |
| MADRS |  |  | -0.167 | 0.426 |
| Inventory of Depressive  Symptomatology | -0.211 | 0.322 |  |  |
| Beck Anxiety Inventory | -0.278 | 0.188 |  |  |
| Fear Questionnaire | -0.115 | 0.592 |  |  |
| Social phobia  subscale | -0.351 | 0.092 |  |  |
| Apathy Scale | -0.068 | 0.753 |  |  |
| Cognitive Failures Questionnaire | -0.257 | 0.226 |  |  |
| Disease duration |  |  | -0.231 | 0.266 |
| Duration of remission |  |  | 0.148 | 0.481 |
| Cushing’s Syndrome  Severity Index |  |  |  |  |
| Active phase | 0.240 | 0.249 |  |  |
| Remission phase | -0.055 | 0.795 |  |  |

MADRS = Montgomery-Åsberg Depression Rating Scale

* = remains significant after Benjamini-Hochberg correction (FDR=20%) for 11 comparisons.
